# Comprehensive analysis of posttranslational protein modifications in aging of subcellular compartments

**DOI:** 10.1038/s41598-020-64265-0

**Published:** 2020-05-05

**Authors:** Tim Baldensperger, Michael Eggen, Jonas Kappen, Patrick R. Winterhalter, Thorsten Pfirrmann, Marcus A. Glomb

**Affiliations:** 1 Institute of Chemistry, Food Chemistry, Martin-Luther-University Halle-Wittenberg, Kurt-Mothes-Str. 2, 06120 Halle/Saale, Germany; 2 Clinic for Heart Surgery, Martin-Luther-University Halle-Wittenberg, Ernst-Grube Str. 40, 06120 Halle/Saale, Germany; 3 Institute of Physiological Chemistry, Martin-Luther-University Halle-Wittenberg, Hollystr. 1, 06114 Halle/Saale, Germany

**Keywords:** Post-translational modifications, Liquid chromatography, Organelles

## Abstract

Enzymatic and non-enzymatic posttranslational protein modifications by oxidation, glycation and acylation are key regulatory mechanisms in hallmarks of aging like inflammation, altered epigenetics and decline in proteostasis. In this study a mouse cohort was used to monitor changes of posttranslational modifications in the aging process. A protocol for the extraction of histones, cytosolic and mitochondrial proteins from mouse liver was developed and validated. In total, 6 lysine acylation structures, 7 advanced glycation endproducts, 6 oxidative stress markers, and citrullination were quantitated in proteins of subcellular compartments using HPLC-MS/MS. Methionine sulfoxide, acetylation, formylation, and citrullination were the most abundant modifications. Histone proteins were extraordinary high modified and non-enzymatic modifications accumulated in all subcellular compartments during the aging process. Compared to acetylation of histone proteins which gave between 350 and 305 µmol/mol leucine equivalents in young and old animals, modifications like acylation, glycation, and citrullination raised to 43%, 20%, and 18% of acetylation, respectively. On the other hand there was an age related increase of selected oxidative stress markers by up to 150%. The data and patterns measured in this study are mandatory for further studies and will strongly facilitate understanding of the molecular mechanisms in aging.

## Introduction

The human genome consists of approximately 20000 genes, which express about 70000 different proteins via alternative splicing. This number is tremendously increased to several million protein species by posttranslational modifications (PTMs)^1^. In comparison to biosynthesis of new proteins, PTMs are formed at much faster rates. Hence, they facilitate rapid adaption of metabolism to environmental changes^2^. One of the most dramatic changes in life is the aging process. Unsurprisingly, hallmarks of aging like mitochondrial dysfunction, inflammation, alteration of epigenetics, and loss of proteostasis are strongly influenced by PTMs^3^.

A key mechanism in gene regulation is the acetylation of lysine residues in histone proteins^4^. Beside regulation of transcription, reversible modification by lysine acetyltransferases and deacetylases is a critical control mechanism in metabolism^5^. Enzymatic citrullination of arginine residues was initially discovered in autoimmune diseases such as rheumatoid arthritis, in which citrullination was increased in inflammatory tissues. This relatively novel modification is formed by peptidyl arginine deiminases. Currently, research focuses on the DNA damage caused by citrullination, which eventually might lead to carcinogenesis^6^.

In contrast to the PTMs described above, a plethora of modifications is formed non-enzymatically. Oxidative stress is a well-known mechanism leading to PTMs which are closely linked to inflammation and impaired proteostasis^7,8^. A long-term research field is the formation of advanced glycation endproducts (AGEs), which are established markers of aging in many extra- and intracellular tissues, e.g., in eye-lens proteins^9^. AGEs predominantly modify lysine and arginine residues of proteins. Previous work identified dysregulation of mitochondrial processes, increased inflammation, and the reduced degradation of proteins by the ubiquitin-proteasome system as results of glycation^10–12^. A special subtype are the amide AGEs, which are lysine acylation structures formed independently from enzymes by glycation^13^. Recently, reactive acyl-CoA species were identified as sources of non-enzymatic lysine acylation, while acylphosphates were hypothesized as a third pathway of non-enzymatic acylation^14^. Due to the structural similarity these lysine acylations are expected to be major alternative pathways to enzymatic acetylation in transcription and metabolic regulation^15^.

Obviously, fundamental important enzymatic regulation of metabolism by PTMs is paralleled by non-enzymatic pathways in aging and disease as reviewed recently^16–18^. To our surprise, no comprehensive data of the various PTMs exists. We previously identified AGEs as excellent markers of aging and disease in cytosolic liver proteins^19^ and identified liver as a local hotspot of protein acylation^20^. Hence, we quantitated 20 different PTMs including 6 lysine acylation modifications, 7 AGEs, 6 oxidative stress markers, and citrulline in histone, mitochondrial, and cytosolic proteins extracted from mice liver using a novel HPLC-MS/MS approach. The relevance of these PTMs in the aging process is discussed on a quantitative basis for the first time. We expect that the described changes of PTMs are key mechanisms in the aging process and will greatly contribute to identify targets for future studies.

## Results

### Fractionation and sample preparation

**Figure 1:**
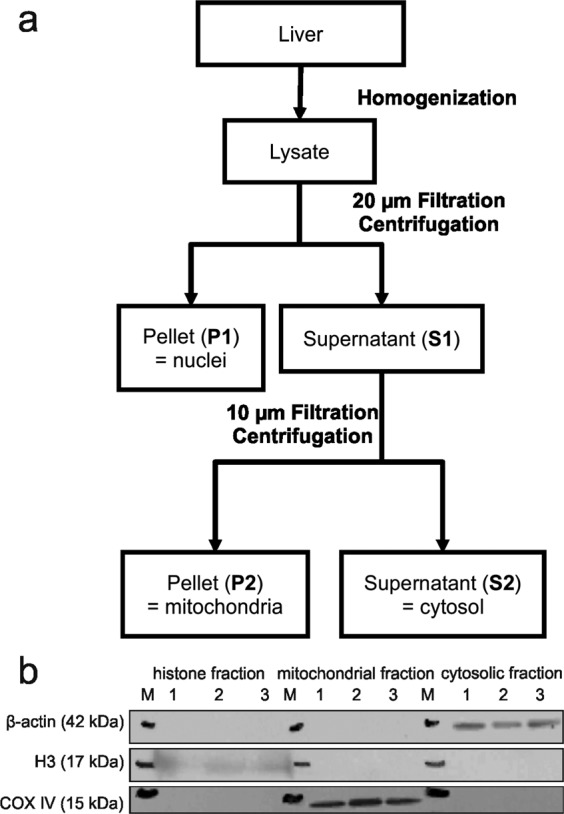
Subcellular fractionation protocol for mice liver (**a**). Western blotting was used to confirm purity of subcellular fractions by detection of cytosolic β‐actin, mitochondrial COX IV, and histone H3 (**b**). M = molecular weight marker. Full-length blots are presented in the supporting information (Figure S1).

A fractionation protocol for mouse liver (Fig. 1a) was developed by combination and optimization of several methods for the isolation of histones^21,22^, mitochondria^23^, and cytosolic proteins^19^. After cell lysis using a tissue grinder in hypotonic sucrose buffer nuclei and mitochondria were separated from cytosol by centrifugation at 800 RCF and 7000 RCF, respectively. The crude fractions were purified by additional clean-up steps like centrifugation for cytosolic fraction, filtration for mitochondria and washing with detergents for nuclei. Finally, mitochondrial proteins were isolated by radioimmunoprecipitation assay buffer (RIPA buffer) and histones were extracted by 0.2 M sulfuric acid. After trichloroacetic acid (TCA) precipitation purity of protein fractions was controlled by Western blotting (Fig. 1b). Antibodies against proteins specific for each subcellular compartment were used, i.e. cytosolic β-actin, mitochondrial COX IV, and histone H3. Markers were exclusively detected in their respective fraction verifying the successful separation using our protocol.

In order to prevent artefact formation proteins were reduced by NaBD_4_ prior to acid and enzymatic hydrolysis as described previously^8^. The average efficiency of enzymatic hydrolysis was about 90%. This was calculated in reference to acid hydrolysis using acid stable *N*^6^-carboxymethyl lysine (CML). Protein hydrolysates were analyzed by HPLC-MS/MS as described under “Methods”. In total, 6 lysine acylation modifications, 7 advanced glycation endproducts (AGEs), 6 oxidative stress markers, and citrulline were quantitated by standard addition calibration using authentic reference standards to cope for matrix influences on, e.g., ionization (Table 1).

**Table 1:** Protein modifications in subcellular compartments of mice liver (mean ± standard deviation, n = 10).

Modifications	[µmol/mol leucine equivalent]					
	Histones		Mitochondria		Cytosol	
	Young (3 month)	Old (24 month)	Young (3 month)	Old (24 month)	Young (3 month)	Old (24 month)
*N*^6^-formyl lysine	57.8 ± 44.5	126.1 ± 44.4*	35.3 ± 13.0	35.6 ± 9.2	17.1 ± 1.7	22.4 ± 4.9*
*N*^6^-acetyl lysine	350.5 ± 119.1	304.5 ± 142.2	43.9 ± 6.9	44.4 ± 13.0	40.1 ± 3.6	44.1 ± 6.3
*N*^6^-propionyl lysine	1.0 ± 0.5	1.7 ± 0.4*	0.7 ± 0.2	0.7 ± 0.3	0.4 ± 0.1	0.7 ± 0.1*
*N*^6^-butyryl lysine	0.3 ± 0.3	0.8 ± 0.3*	0.2 ± 0.1	0.3 ± 0.1*	0.1 ± 0.1	0.3 ± 0.1*
*N*^6^-malonyl lysine	<LOD	< LOD	2.6 ± 0.4	2.3 ± 0.4	2.9 ± 0.4	3.5 ± 0.6*
*N*^6^-succinyl lysine	2.1 ± 0.3	2.7 ± 0.4*	4.2 ± 0.7	4.2 ± 1.9	0.4 ± 0.1	0.6 ± 0.1*
CML	13.5 ± 3.9	22.1 ± 7.0*	4.5 ± 0.7	7.2 ± 2.1*	6.1 ± 1.1	7.5 ± 1.3*
GALA	1.2 ± 0.3	1.8 ± 0.4*	0.4 ± 0.1	0.5 ± 0.2*	0.3 ± 0.1	0.4 ± 0.1*
G-H3	15.7 ± 4.8	22.6 ± 8.5*	14.9 ± 4.3	24.1 ± 11.6*	23.4 ± 10.6	40.9 ± 6.4*
CEL	2.7 ± 0.4	3.4 ± 0.6*	3.1 ± 0.6	4.1 ± 0.6*	13.0 ± 2.8	15.6 ± 3.3*
*N*^6^-lactoyl lysine	<LOD	< LOD	0.2 ± 0.1	0.3 ± 0.1*	0.1 ± 0.1	0.2 ± 0.1*
MG-H	6.7 ± 2.4	8.6 ± 2.1*	5.6 ± 0.7	7.0 ± 1.0*	15.0 ± 3.9	24.3 ± 13.2*
furosine	2.5 ± 0.2	3.1 ± 1.1*	0.6 ± 0.5	0.9 ± 0.9	2.9 ± 0.7	2.7 ± 0.7
*N*^6^-glyoxylyl lysine	0.4 ± 0.2	0.8 ± 0.2*	<LOQ	< LOQ	< LOQ	< LOQ
*N*^6^-pyruvoyl lysine	0.8 ± 0.3	1.5 ± 0.3*	<LOQ	< LOQ	< LOQ	< LOQ
*o*-tyrosine	0.7 ± 0.2	1.1 ± 0.3*	4.4 ± 0.8	5.8 ± 0.9*	3.6 ± 1.0	6.1 ± 1.4*
*o,o*-dityrosine	2.1 ± 0.4	4.7 ± 2.4*	<LOQ	< LOQ	< LOQ	< LOQ
methionine sulfoxide	704.9 ± 323.2	595.8 ± 251.0	2454.9 ± 1084.2	1449.3 ± 767.0	1200.4 ± 777.7	1845.1 ± 770.3*
methionine sulfone	64.8 ± 42.6	104.9 ± 27.7*	41.3 ± 22.1	40.9 ± 21.1	20.9 ± 16.6	39.0 ± 21.5*
citrulline	37.6 ± 13.9	55.2 ± 16.2*	27.9 ± 16.4	30.0 ± 12.4	5.3 ± 1.8	5.7 ± 1.3

Significant differences (unpaired t-test, p < 0.05) between young and old animals (n = 10) are indicated by an asterisk. CML = *N6*-carboxymethyl lysine; GALA = *N6*-glycoloyl lysine, G-H3 = glyoxal hydroimidazolone 3; CEL = *N6*-carboxyethyl lysine; MG-H = methylglyoxal hydroimidazolone.

### Acylation

**Figure 2:**
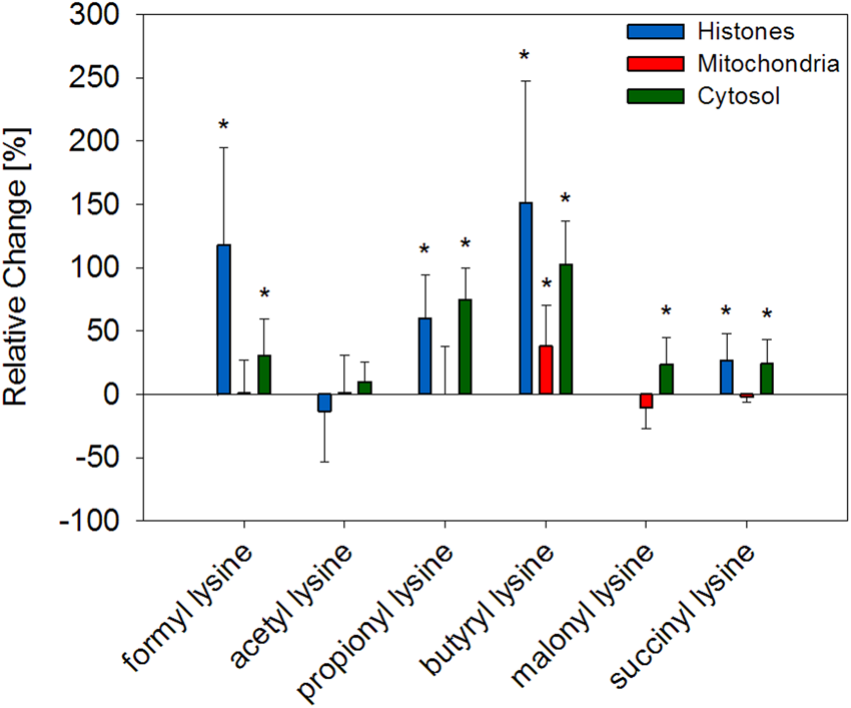
Changes of acylation in aging mice liver. Relative changes of mean modification levels in old mice were compared to mean levels in young mice. Error bars represent relative standard deviation of old animals. Significant differences (unpaired t-test, p < 0.05) between young and old animals (n = 10) are indicated by an asterisk.

Enzymatically formed acetylation and non-enzymatic lysine acylation were quantitated in subcellular fractions of liver (Table 1) in the aging process (Fig. 2).

Here, 3 month old C57BL/6 N mice (n = 10) were compared to 24 month old animals (n = 10). Acetylation was the most abundant lysine modification in mice liver with average concentrations between 40.1 and 44.4 µmol/mol leucine equivalents (leucine-eq) in cytosolic as well as in mitochondrial proteins. Especially high concentrations were measured in histone proteins, in which *N*^6^-acetyl lysine concentrations were approximately 10 times higher (350.5 µmol/mol leucine-eq) compared to cytosol and mitochondria. Despite the modification’s pivotal role in epigenetics and metabolic regulation no correlation with aging was observed.

Formylation was the second most abundant lysine modification. Again, levels of *N*^6^-formyl lysine were highest in histones (126.1 µmol/mol leucine-eq) and by a factor of 6 lower in cytosolic proteins (22.4 µmol/mol leucine-eq). This trend is in line with the lower abundant aliphatic acylation structures *N*^6^-propionyl lysine with 1.7 µmol/mol leucine-eq in histones vs. 0.7 µmol/mol leucine-eq in cytosol and *N*^6^-butyryl lysine with 0.8 µmol/mol leucine-eq in histones vs. 0.3 µmol/mol leucine-eq in cytosol. In contrast to acetylation aliphatic acylations increased about 50% in aged histones and cytosolic proteins with p-values ranging between 0.005 and 0.001. Another picture emerged for mitochondrial formylation and propionylation. While concentrations of 35.6 µmol/mol leucine-eq for *N*^6^-formyl lysine and 0.7 µmol/mol leucine-eq for *N*^6^-propionyl lysine were in between histone and cytosolic fraction, no age dependent increase was detected in mitochondria. On the other hand, butyrylation significantly increased (p = 0.020) with aging in mitochondria.

The acidic lysine acylations *N*^6^-malonyl and *N*^6^-succinyl lysine were especially high abundant in mitochondria with 2.3 and 4.2 µmol/mol leucine-eq, respectively. Succinylation was lowest in cytosolic proteins and malonylation was below limit of quantitation (LOD) in histones. Again, no correlation with aging was observed in mitochondria but concentrations of both structures increased significantly about 50% (p < 0.006) in aged histones and cytosolic proteins.

### Glycation

**Figure 3:**
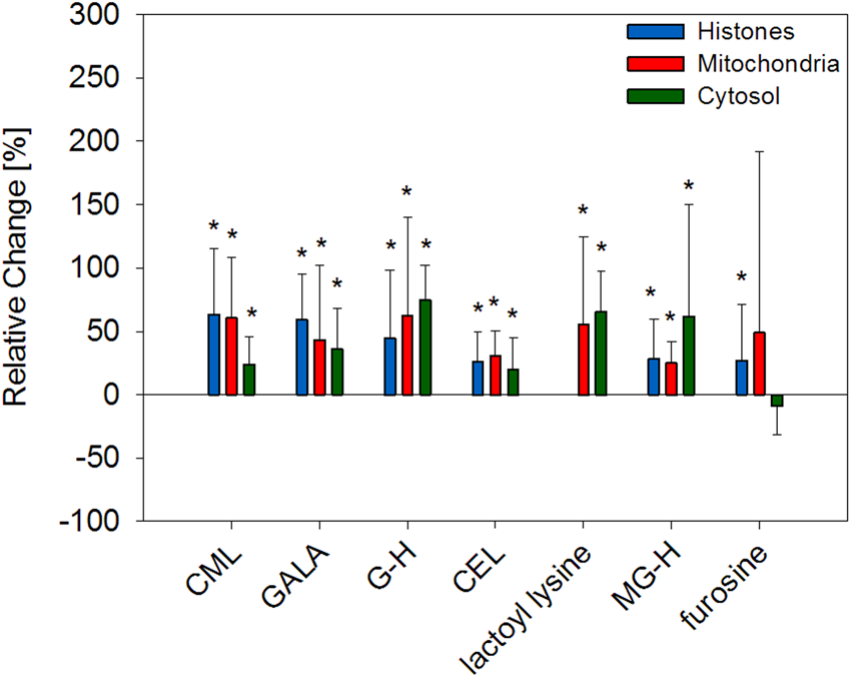
Changes of advanced glycation endproducts in aging mice liver. Relative changes of mean modification levels in old mice were compared to mean levels in young mice. Error bars represent relative standard deviation of old animals. Significant differences (unpaired t-test, p < 0.05) between young and old animals (n = 10) are indicated by an asterisk.

Advanced glycation endproducts (AGEs) accumulated on average 50% during aging (Fig. 3, Table 1).

Furosine as a marker of early stage lysine glycation by Maillard reactions was low abundant (0.9 µmol/mol leucine-eq) in mitochondria. Concentrations were a factor of 3–5 higher in histones (3.1 µmol/mol leucine-eq) and cytosolic (2.7 µmol/mol leucine-eq) proteins. Contrary to all end-stage Maillard glycation AGEs measured herein, no age dependent increase was observed for furosine in mitochondria and cytosol but the structure significantly (p = 0.044) correlated with aging of histones.

Quantitative more important were the AGEs formed by short-chained α-dicarbonyls glyoxal and methylglyoxal. CML was extraordinary high abundant in histones (22.1 µmol/mol leucine-eq) and, thus, more than twice as high concentrated as in mitochondrial and cytosolic proteins. In contrast, *N*^6^-carboxyethyl lysine (CEL) as the corresponding lysine modification by methylglyoxal was concentrated 5 times higher in cytosol compared to histones with 15.6 vs. 3.4 µmol/mol leucine-eq, respectively. The glyoxal specific amide AGE *N*^6^-glycoloyl lysine (GALA) indicated the same trend. GALA concentrations were about 3 times higher in histones compared to mitochondria and cytosol. Levels of methylglyoxal specific *N*^6^-lactoyl lysine were below LOD in histones and around 0.2 µmol/mol leucine-eq in mitochondria and cytosol. Glyoxal hydroimidazolone (G-H) as a measure for glyoxal-arginine AGEs and methylglyoxal hydroimidazolone (MG-H) were quantitative important modifications in all three compartments. Compared to lysine AGEs the differences between fractions were smaller, but average glycation levels of arginine were higher. Overall, modifications by short-chained α-dicarbonyls and aging correlated in all compartments with p-values between 0.001 and 0.043.

### Oxidative stress

**Figure 4:**
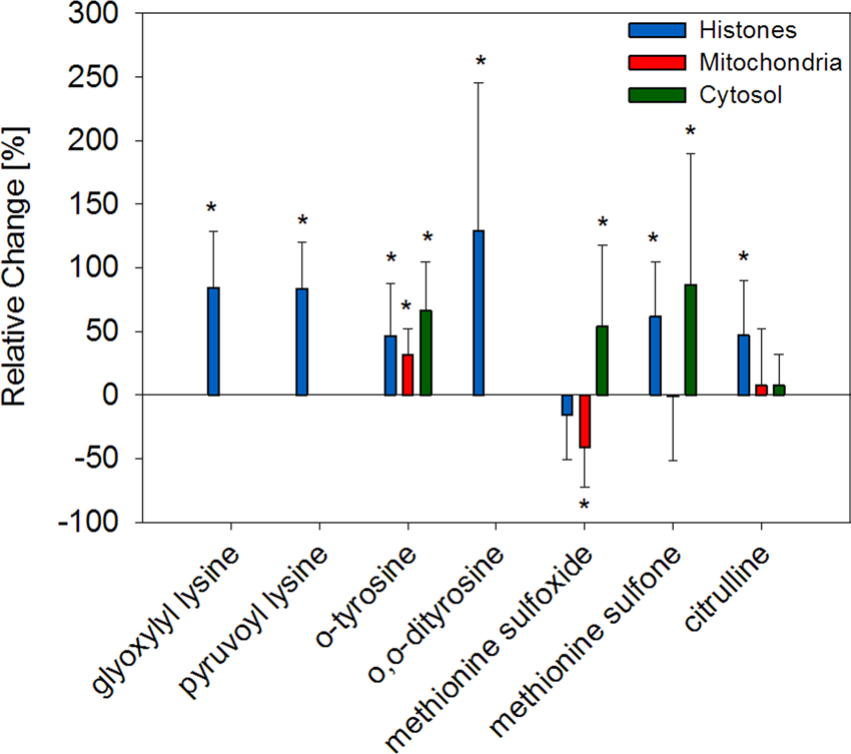
Changes of oxidative stress markers and citrullination in aging mice liver. Relative changes of mean modification levels in old mice were compared to mean levels in young mice. Error bars represent relative standard deviation of old animals. Significant differences (unpaired t-test, p < 0.05) between young and old animals (n = 10) are indicated by an asterisk.

Markers of oxidative stress were quantitated in liver fractions **(**Table 1**)** of the aging mice cohort (Fig. 4).

The α-oxoamide AGEs *N*^6^-glyoxylyl and *N*^6^-pyruvoyl lysine are formed by glycation under oxidative conditions from glyoxal and methylglyoxal, respectively. Thus, they are markers of carbonyl stress as well as oxidative stress^19^. Concentrations of *N*^6^-glyoxylyl lysine equalled 30% of corresponding α-hydroxyamide AGE GALA in histones of young mice (0.4 µmol/mol leucine-eq) and increased to 100% in old mice (0.8 µmol/mol leucine-eq). *N*^6^-pyruvoyl lysine levels rose up in a similar way from 0.8 to 1.5 µmol/mol leucine-eq (p < 0.001). Unfortunately, *N*^6^-glyoxylyl and *N*^6^-pyruvoyl lysine were below LOQ in other fractions.

Oxidatively dimerized *o,o*-dityrosine is another confirmed oxidative stress marker, which was below LOQ in mitochondria and cytosol, but rather high abundant in histones. Moreover, significant effects (p = 0.003) were observed during aging with concentrations approximately twice as high at 4.7 µmol/mol leucine-eq in old animals. Oxidation of phenylalanine by hydroxyl radicals leads to *o*-tyrosine. This oxidative stress marker was detected in all analyzed compartments and was especially high concentrated in mitochondria and cytosol with up to 5.8 and 6.1 µmol/mol leucine-eq, respectively. On the other hand, in histones about 25% of the amount determined in the other fractions was found. Nevertheless, *o-*tyrosine was an excellent marker of aging with p-values below 0.008.

Methionine sulfoxide was the most abundant modification measured in the present study. Concentrations ranged between 2455 µmol/mol leucine-eq in mitochondria and 596 µmol/mol leucine-eq in histones. The development of methionine sulfoxide levels in aging was very different between analyzed subcellular fractions. While no significant trend was determined in histones, methionine sulfoxide significantly decreased in mitochondria (p = 0.015) and increased in cytosol (p = 0.039). In general, irreversible further oxidation from methionine sulfoxide to methionine sulfone occurred on a relatively low stoichiometry of about 2%. Nevertheless, about 10–20% oxidation to methionine sulfone was detected in histone proteins, which significantly increased in the aging process (p = 0.013).

### Citrullination

The most abundant arginine modification in histones was enzymatic citrullination (Table 1). Concentrations reached up to 55.2 µmol/mol leucine-eq and were by a factor of 10 lower in cytosol. In mitochondria average values of about 30 µmol/mol leucine-eq were detected. While no correlation with aging was observed in mitochondrial and cytosolic proteins, a significant increase (p = 0.010) was measured in histone proteins (Fig. 4).

## Discussion

Acetylation as the most abundant lysine modification is a key mechanism in regulation of transcription and metabolism^5^. Consequently, especially high concentrations of *N*^6^-acetyl lysine were found in histone protein, in which acetylation modifies chromatin structures and, thus, accessibility of the DNA for, e.g., transcription. Due to the strict enzymatic regulation by acetyltransferases and deacetylases no changes were detected in the aging process in histones, but also for proteins in the other subcellular fractions. This latter observation was in line with previously published studies in rat liver mitochondria and human eye lens proteins^9,24^. However, for a total assessment of the concept of acetylation it has to be kept in mind that central intermediates of Maillard triggered glucose degradation also result in lysine acetylation. Specifically, *in vitro* incubations of 1-deoxyglucosone resulted in up to 65% acetic acid and related acetylation products via hydrolytic β-dicarbonyl cleavage^25,26^. This might also explain, why in case of histone acetylation, interrelationships are complex and contradictory trends were reported depending on organism, tissue, site-specific position etc^27,28^. Although no change of total *N*^6^-acetyl lysine levels in mouse liver was detected in the present study, non-proliferating organs like brain and site specific analysis of acetylation remain important topics in the field of aging research.

Acetylation is paralleled by further structurally related non-enzymatic acylations via Maillard reactions, reactive acylphosphates, and acyl-CoA species^14,26,29^. For the latter, enzymatic acylation by promiscuous acyltransferase activities of several lysine acetyltransferases was discussed in recent publications. However, it has to be considered that these enzymatic activities are magnitudes lower than corresponding acetyltransferase activities^15^. Independent from origin, our data suggests that acylation is a major alternative pathway to lysine acetylation. While total acylation reached approximately 50% of *N*^6^-acetyl lysine levels in histones and cytosol almost equivalent amounts of acylation and acetylation were found in mitochondria. Acylation was an extraordinary good marker of aging in histones and cytosolic proteins with p-values below 0.005. Research on the biological impact of these lysine modifications is very likely mandatory for a better understanding of epigenetic and metabolic dysregulation in aging and related fields. In contrast, mitochondrial acylation indicated no effects in aging despite high abundance. A possible explanation is the presence of mitochondrial deacylases. Specifically, sirtuins 3–5 act on sites of propionylation, malonylation, and succinylation^30,31^. Obviously, this enzymatic control system maintains constant acylation levels during aging of mitochondria. The single exception from this observation was the increase of low abundant butyrylation, which is not targeted by any mitochondrial sirtuin^31^. Acidic acylations *N*^6^-malonyl and *N*^6^-succinyl lysine are closely linked to energy metabolism (citric acid cycle and fatty acid synthesis). Hence, they are considered as regulatory motives^32,33^. Previous work supports our findings, because it was shown that succinylation is not increasing with age in mammalian mitochondria but in *C*. *elegans* and *D*. *melanogaster* which are lacking any sirtuin 5 homologues^24^.

An impact of sirtuins on formylation has not been reported so far, although only mitochondrial proteins showed no age correlation in the present study. Formylation was next to acetylation by far the most abundant acylation structure detected. First, formylation was described to be initiated by oxidative DNA degradation via a hypothetical formyl phosphate intermediate leading to changes in chromatin structure^29,34^. Later, formaldehyde metabolism emerged as a possible source of formylation^35^. In addition, Maillard glycation was verified as a source of lysine formylation. In parallel to the above fragmentation of 1-deoxyglucosone leading to acetylation, here fragmentation of glucosone was verified as the origin^36^. Despite the high abundance and strikingly clear correlation with aging, the physiological relevance of formylation remains poorly understood and further research in this field is mandatory.

It has to be stated that non-enzymatic glycation beside formylation and acetylation leads to many more acylation products collectively termed as amide AGEs, e.g., amides of glycolic acid (GALA), lactic acid, glyceric acid, oxalic acid, glyoxylic acid and pyruvic acid^37^. An established marker of early stage Maillard reaction is the Amadori product which can be accessed after acid hydrolysis as furosine^38^. Furosine was the only modification formed by glycation which was not increasing with age of mitochondrial and cytosolic proteins. In contrast, furosine accumulated in aged histones. Possible explanations for this apparent contradiction are the continuous formation and fragmentation of the Amadori product as an important reactive Maillard intermediate as well as the prolonged protein half-time of 117 days of histones from mice liver^39^. Quantitative more important was the glycation by short-chained α-dicarbonyl compounds glyoxal and methylglyoxal, which are reactive intermediates generated *in vivo* not only by Maillard degradation of sugars as glucose, but additionally stem to a major extend from lipoxidation and glycolysis^40^. Through complex isomerization cascades glyoxal leads to lysine modifications CML and GALA while corresponding structures CEL and *N*^6^-lactoyl lysine are formed by methylglyoxal^19^. A particularly interesting observation herein was the relative accumulation of methylglyoxal specific CEL and *N*^6^-lactoyl lysine especially in cytosol and mitochondria. This highlights the importance of triose phosphates from cytosolic glycolysis as important methylglyoxal precursors *in vivo*^40^. This notion is further supported by the highest concentrations found in cytosol for MG-H, which is a methylglyoxal specific arginine modification. Here, the values for MG-H are given as a sum for the two isomeric forms MG-H1 and MG-H3. The higher values of MG-H compared to CEL measured in all subcellular fractions is in line with the higher reactivity of the guanidine function of arginine versus the *N*^6^-amino function of lysine towards α-dicarbonyl compounds^8^. In contrast, the glyoxal specific hydroimidazolinone G-H3 is an artefact of acid protein hydrolysis but has been shown to be a useful quantitative tool for glyoxal-dihydroxyimidazolidine and the AGE *N*^7^-carboxymethyl arginine^41^. Obviously, glyoxal induced glycation increased in all cell fractions with age. The same was true for CML and GALA with exceptionally high values of 22.1 µmol/mol leucine-eq for CML in histones. In contrast to GALA and glyoxal arginine modifications it has to be considered, that there are major alternative pathways for CML formation. Notably, the oxidative fragmentation of the Amadori product which makes CML also a parameter of oxidative stress^8^. In total, AGEs were excellent markers of aging in all subcellular compartments. Beside correlation with aging several studies express causal relationships between AGEs and changed chromatin structure^42^, mitochondrial dysfunction^10^, loss of proteostasis^43^, as well as inflammation^44^.

The formation of α-oxoamide AGEs *N*^6^-glyoxylyl and *N*^6^-pyruvoyl lysine in the CML/CEL reaction cascades by oxidation of an enaminol intermediate represents a combination of carbonyl and oxidative stress^19^. Unfortunately, concentrations of α-oxoamide AGEs were below LOQ in mitochondrial and cytosolic proteins. In histones both α-oxoamide AGEs increased in aging as measured for all other AGEs. In addition, the ratio between oxidatively formed *N*^6^-glyoxylyl lysine and corresponding non-oxidatively formed GALA changed from about 0.3 in young to 0.5 in old animals. Both AGEs share a common enaminol precursor and a shift from α-hydroxyamide to α-oxoamide AGE thus clearly indicated increased oxidative stress in aged animals. The established oxidative stress markers *o-*tyrosine and *o,o-*dityrosine confirmed the increase of oxidative stress with aging. While *o-*tyrosine is formed by oxidation of phenylalanine residues via hydroxyl radicals *o,o-*dityrosine is generated by cross-linking of tyrosine residues either by hydroxyl radicals or tyrosyl radicals^45^. Methionine sulfoxide was by far the most abundant modification detected in the present paper reaching up to 2500 µmol/mol leucine-eq. According to literature methionine oxidation to the sulfoxide has a pivotal role in maintaining the cellular redox state by operating as an oxidative sink^46^. One could assume that the herein proven increase of age associated oxidative stress should result in an increase of methionine sulfoxide concentration. It is important to understand that the oxidation of methionine is an equilibrium, which is regulated by methionine sulfoxide reductases. Consequently, methionine sulfoxide is no stable accumulative modification, but rather a snapshot of current cellular redox state and working antioxidative defence mechanisms. In contrary, further oxidation of methionine sulfoxide to methionine sulfone is an irreversible step resulting in an age dependent increase in histones and cytosol. To our knowledge we were the first group quantitating methionine sulfone *in vivo* via our highly sensitive HPLC-MS/MS approach.

Enzymatic citrullination is closely linked to inflammatory processes and was the most abundant arginine modification in histones and mitochondria, while in cytosolic proteins glyoxal and methylglyoxal arginine AGEs prevailed by a factor of 10. In addition, concentration of citrullination was exclusively increasing with aging in histones. This is especially fatal, because citrullination is believed to activate DNA damaging pathways leading to carcinogenesis^6^.

In summary, non-enzymatic posttranslational modifications were accumulating in all subcellular compartments in aging. On the other hand, the relative proportion of enzymatic acetylation among all detected modifications (excluding methionine sulfoxide) decreased from 60 to 45% in histones with age and from about 30% to 20% in mitochondria and cytosol. This decrease was mainly caused by non-enzymatic acylation rising to 43% of *N*^6^-acetyl lysine concentration in histone proteins. In addition, glycation, oxidative stress markers and citrullination increased to 20%, 37% and 18% of acetylation levels during aging, respectively. Glycation was the single most important factor for the relative decrease of acetylation in aging mitochondria and increased to equal amounts compared to acetylation. Acylation, oxidative stress markers and citrullination were measured approximately in the same concentration, but no correlations with aging were detected in mitochondria. In cytosolic proteins of old mice glycation tremendously increased to 200% of *N*^6^-acetyl lysine. While levels of citrullination were constant in cytosolic proteins, acylation and oxidative stress markers increased to 62% and 100% of acetylation, respectively. Finally, we postulate that comprehensive analysis of changes in protein modification patterns presented in this study will be mandatory to understand the molecular mechanisms of aging.

## Methods

### Chemicals and Enzymes

All chemicals of the highest quality available were provided by Sigma-Aldrich (Munich/Steinheim, Germany), unless otherwise indicated. Pronase E was purchased from Sigma-Aldrich. Carboxypeptidase Y and leucine aminopeptidase were prepared as described previously^47,48^. *N*^6^-formyl lysine, *N*^6^-acetyl lysine, furosine, methionine sulfoxide, methionine sulfone, *o*-tyrosine, *o,o*-dityrosine, and citrulline were purchased from Sigma-Aldrich. Synthesis and structure elucidation of commercially unavailable authentic reference standards was described previously^9,13,19,20^. Structural formulas of analytes are included in the supporting information (Figure S2).

### Housing of animals

Male C57BL/6 N mice were purchased at the age of 6 weeks from Janvier Laboratories (Le Genest-Saint-Isle, France) and housed in individually ventilated cage systems in a climate room (20.3 °C, 62% humidity) with 12 hours circadian rhythm and fed ad libitum under barrier conditions in the Center of Medical Basic Research at the Medical Faculty (University of Halle, Germany). The tissues were collected at the age of 3 month (young) or 24 month (old). All work on the mice was performed at a sterile workbench in the same room. The principles for the care and use of animals from the American Physiological Society guide were followed. The announcement to kill vertebrates for scientific purposes for this tissue collection was approved by the local authority (K2BM3, MLU Landesverwaltungsamt, Sachsen-Anhalt, Germany).

### Tissue collection

The mice were anaesthetized with 100 mg/kg bodyweight ketamine (Zoetis, Berlin, Germany) and 10 mg/kg bodyweight Xylazine (Bayer, Leverkusen, Germany). The animals were killed in deep narcosis by subluxation of cervical spine. The livers were dissected and immediately snap-frozen on dry ice.

### Fractionation

The developed fractionation protocol (Fig. 1a) is a modified combination of several methods for the isolation of histones^21,22^, mitochondria^23^ and cytosolic proteins^19^.

All steps were performed at 4 °C using pre-cooled lab ware and solutions. About 300 mg mice liver was rinsed free of blood by repetitive washing in phosphate buffered saline. The liver was homogenized in 600 µL homogenization buffer (0.32 M sucrose, 3 mM MgCl_2_, 10 mM nicotine amide, 500 nM trichostatin A) supplemented with EDTA-free protease inhibitor mixture (Roche, Pleasanton, USA) using a disposable tissue grinder (VWR International, Radnor, USA). Cell debris was removed by 20 µm Celltrics filtration (Sysmex, Norderstedt, Germany). Nuclei were pelleted (**P1**) by centrifugation at 800 RCF for 15 min. The supernatant (**S1**) was decanted and purified by 10 µm Celltrics filtration. After centrifugation at 7000 RCF for 15 min the resulting supernatant (**S2**) containing cytosolic proteins was separated from the pellet (**P2**) containing mitochondria.

### Extraction histone proteins

Nuclear Pellet (**P1**) was washed using 1 mL nuclear washing buffer (0.35 M sucrose, 0.5% Triton-X100, 10 mM KCl and 1.5 mM MgCl_2_ in 10 mM HEPES buffer pH 7.4) and a second time using 1 mL nuclear washing buffer without Triton-X100. Centrifugation at 800 RCF for 15 min pelleted purified nuclei. Histones were extracted from this pellet in 400 µL 0.2 M H_2_SO_4_ by a MM 400 mixer mill (Retsch, Haan, Germany) for 15 min and 1 h incubation on ice. Debris was removed by centrifugation at 16100 RCF for 15 min. The resulting supernatant contained purified histone proteins.

### Extraction mitochondrial proteins

Mitochondrial pellet (**P2**) was washed twice using 1 mL mitochondrial washing buffer (0.2 M sucrose and 1 mM EDTA in 10 mM Tris buffer pH 7.4) and centrifuged at 7000 RCF for 15 min. Purified mitochondria were lysed in 400 µL RIPA buffer (150 mM NaCl, 1 mM EDTA, 0.25% deoxycholate, 1% Nonidet P-40, and 1% sodium dodecylsulfate in 50 mM Tris-HCl buffer pH 7.4) by a MM 400 mixer mill for 15 min and 2 h incubation on ice. Debris was removed by centrifugation at 16100 RCF for 15 min. The resulting supernatant contained purified mitochondrial proteins.

### Extraction cytosolic proteins

Remaining mitochondria were removed from supernatant (**S2**) by additional centrifugation at 7000 and 16100 RCF for 15 min until no pellet formation was observed. The resulting supernatant contained purified cytosolic proteins.

### Protein work-up

Proteins were precipitated at 10% trichloroacetic acid concentration by addition of 50% stock solution and centrifugation at 1000 RCF for 15 min. Pellets were washed 3 times using 80% ice-cold acetone and allowed to air-dry. Proteins were dissolved in 500 µL 0.1 M Tris buffer (pH 7.4) and homogenized by a MM 400 mixer mill. Protein concentrations were adjusted to a maximum of 3 mg/mL. Addition of 100 µL NaBD_4_ solution (15 mg/mL in 0.01 M NaOH) and reduction for 1 h at room temperature was used to prevent artefact formation. Excess of NaBD_4_ was destroyed by addition of 100 µL 1 M HCl and neutralization by 100 µL 1 M NaOH.

### Western blotting

Proteins were extracted as described above. Total protein amount was determined by Lowry assay and 10 μg were loaded per lane. Novex Wedge Well 8–16% gradient gels (Invitrogen, Carlsbad, USA) and 0.45 µm Amersham Hybond PVDF membranes (GE Healthcare, Amersham, United Kingdom) were used. Primary antibodies (mouse anti β‐actin, 1:5000, Sigma #A5441; mouse anti COX IV, 1:5000, Abcam #ab33985; rabbit anti H3, 1:1000, Abcam #ab1791) were applied, followed by HRP‐conjugated secondary antibodies and Super Signal West Pico Plus chemiluminescence kit (Thermo Fisher Scientific, Waltham, MA), according to manufacturer instructions. Signals were detected by X-ray films and scanned in the transparency mode.

### Enzymatic hydrolysis

Enzymatic hydrolysis was performed as described previously^20^. A small crystal of thymol was added to aliquots (250 µL) of protein solutions. Enzymes were added stepwise starting with 30 µL pronase E (0.3 units), 10 µL carboxypeptidase Y (0.1 units) after 48 h, and 10 µL leucine aminopeptidase (0.5 units) after 72 h. Samples were incubated at 37 °C in a shaker incubator for 96 h. Once the total digestion procedure was completed, reaction mixtures were filtered through 3 kDa molecular weight cut-off filters (VWR International, Radnor, USA).

### Acid hydrolysis

Acid hydrolysis was performed as described previously^13^. Aliquots of protein solutions (250 μL) were dried in a vacuum concentrator (Savant-Speed-Vac Plus SC 110 A combined with a Vapor Trap RVT 400, Thermofisher Scientific, Bremen, Germany). 800 µL of 6 M HCl was added and the solution was heated 20 h at 110 °C under an argon atmosphere. Volatiles were removed in a vacuum concentrator and the residue was dissolved in 300 µL of ultra-pure water. Samples were filtered through 0.45 μm cellulose acetate Costar SpinX filters (Corning Inc., Corning, USA). Acid stable structures furosine, CML, CEL, G-H, methionine sulfoxide, methionine sulfone, *o*-tyrosine, and *o,o*-dityrosine were quantitated from acid hydrolysates, all other analytes from enzymatic hydrolysates.

### Ninhydrin assay

After complete workup the amount of amino acids in hydrolysates was determined by ninhydrin assay and referenced to a calibration of l-leucine concentrated between 5 and 100 µM as described previously^9^. The absorbance was determined at 546 nm with an Infinite M200 microplate reader (Tecan, Männedorf, Switzerland) using 96-well plates. Each sample was prepared three times.

### Analytical HPLC-MS/MS

A PU-2080 Plus quaternary gradient pump with degasser and a AS-2057 Plus autosampler (Jasco, Gross-Umstadt, Germany) were used as described previously^9^. The mass analyses were performed using an API 4000 quadrupole instrument (Applied Biosystems, Foster City, USA) equipped with an API source using electrospray ionization. The HPLC system was connected directly to the probe of the mass spectrometer. Nitrogen was used as sheath and auxiliary gas. To measure the analytes the scheduled multiple-reaction monitoring (sMRM) mode of HPLC-MS/MS was used. The optimized parameters for mass spectrometry are given in Table 2.

**Table 2:** Mass spectrometric parameters for quantitation.

Modification	Retention time (min)	Precursor		Product ion 1^a^			Product ion 2^b^			Product ion 3^b^		
		m/z (amu)	DP (V)	m/z (amu)	CE (eV)	CXP (V)	m/z (amu)	CE (eV)	CXP (V)	m/z (amu)	CE (eV)	CXP (V)
**Acylation**												
*N*^6^-formyl lysine	11.5	175.1	40	112.1	20	13	84.1	35	7	129.1	15	13
*N*^6^-malonyl lysine	13.4	233.2	45	126.2	20	10	84.2	38	10	170.3	22	12
*N*^6^-acetyl lysine	14.7	189.2	40	126.1	18	10	84.2	31	5	143.1	14	10
*N*^6^-succinyl lysine	16.8	247.1	50	84.3	40	10	184.4	21	12	130.1	26	12
*N*^6^-propionyl lysine	19.1	203.0	45	84.3	35	12	140.4	19	13	157.4	15	10
*N*^6^-butyryl lysine	23.3	217.0	50	84.3	32	5	154.3	18	10	171.4	17	10
**Glycation**												
*N*^6^-glycoloyl lysine	10.4	205.2	40	142.1	20	11	84.1	36	14	56.2	64	8
*N*^6^-carboxymethyl lysine	9.6	205.1	42	130.2	17	9	84.1	30	14	56.1	59	9
*N*^6^-lactoyl lysine	14.3	219.2	40	156.2	20	8	84.1	35	9	173.1	17	8
*N*^6^-carboxyethyl lysine	14.4	219.1	54	84.1	33	7	130.1	18	11	56.1	59	8
glyoxal hydroimidazolone	17.3	215.1	48	100.1	20	8	70.1	38	11	116.2	20	10
methylglyoxal hydroimidazolone	23.0	229.2	45	114.2	21	9	70.2	45	11	116.1	21	9
furosine	24.4	255.2	50	84.1	33	7	130.3	21	10	192.0	23	12
**Oxidative Stress**												
methionine sulfone	4.4	182.2	45	136.0	16	9	56.2	35	8	—	—	—
methionine sulfoxide	4.5	165.9	33	74.2	20	12	56.1	30	10	102.1	19	18
*N*^6^-glyoxylyl lysine	10.4	206.2	40	143.1	20	11	84.1	36	14	160.1	15	13
*N*^6^-pyruvoyl lysine	14.3	220.2	40	157.2	20	8	84.1	35	9	174.1	30	15
*o*-tyrosine	25.3	182.1	45	136.2	18	11	91.1	42	15	119.1	27	10
*o,o*-dityrosine	27.9	361.2	25	315.3	23	7	254.2	33	15	237.2	34	21
**Citrullination**												
citrulline	5.5	176.2	40	159.2	14	11	70.3	35	11	113.1	22	7

^a^MRM transition used for quantitation. ^b^MRM transition used for confirmation.

Quantitation was based on the standard addition method using known amounts of the pure authentic reference compounds to compensate for matrix effects. Authentic reference compounds were added at 0.5, 1, 2, and 4 times the concentration of the analyte in the sample and correlation coefficients were 0.9 or higher. Limits of detection and quantitation are given in Table 3.

**Table 3:** Limits of detection (LOD) and limits of quantitation (LOQ).

Modifications	[µmol/mol leucine equivalent]					
	Histones		Mitochondria		Cytosol	
	LOD	LOQ	LOD	LOQ	LOD	LOQ
*N*^6^-formyl lysine	1.1	3.8	1.8	6.0	2.5	8.3
*N*^6^-acetyl lysine	0.1	0.4	0.5	1.8	0.7	2.3
*N*^6^-propionyl lysine	0.1	0.4	0.2	0.6	0.1	0.4
*N*^6^-butyryl lysine	0.04	0.1	0.02	0.07	0.02	0.07
*N*^6^-malonyl lysine	0.9	2.9	0.3	0.9	0.4	1.3
*N*^6^-succinyl lysine	0.4	1.4	0.03	0.1	0.04	0.1
CML	0.4	1.2	0.5	1.7	0.7	2.4
GALA	0.3	0.9	0.1	0.3	0.1	0.2
G-H3	0.6	2.0	1.0	3.4	1.1	3.6
CEL	0.4	1.3	0.5	1.8	0.5	1.8
*N*^6^-lactoyl lysine	0.3	1.0	0.1	0.2	0.03	0.1
MG-H	0.7	2.4	0.3	1.1	0.6	1.9
furosine	0.1	0.4	0.04	0.1	0.05	0.2
*N*^6^-glyoxylyl lysine	0.1	0.4	0.3	0.8	0.4	1.5
*N*^6^-pyruvoyl lysine	0.1	0.3	0.3	0.9	0.4	1.4
*o*-tyrosine	0.1	0.2	0.2	0.6	0.1	0.4
*o,o*-dityrosine	0.4	1.4	0.4	1.3	0.5	1.7
methionine sulfoxide	0.1	0.2	0.2	0.7	0.2	0.8
methionine sulfone	0.6	2.0	0.6	2.2	2.8	9.3
citrulline	0.4	1.3	0.4	1.2	0.3	0.9

CML = *N*^6^-carboxymethyl lysine; GALA = *N*^6^-glycoloyl lysine, G-H3 = glyoxal hydroimidazolone 3; CEL = *N*^6^-carboxyethyl lysine; MG-H = methylglyoxal hydroimidazolone.

Chromatographic separations were performed on a stainless steel column (XSelect HSS T3, 250×3.0 mm, RP18, 5 µm, Waters, Milford, USA) using a flow rate of 0.7 mL/min and a column temperature of 25 °C. Eluents were ultra-pure water (A) and a mixture of methanol and ultra-pure water (7:3, v/v; B), both supplemented with 1.2 mL/L heptafluorobutyric acid. Samples were injected (10 µL) at 2% B and run isocratic for 2 min, gradient was changed to 14% B within 10 min (held for 0 min), 87% B within 22 min (held for 0 min), 100% B within 0.5 min (held for 7 min) and 2% B within 2.5 min (held 8 min). Exemplary chromatographic separations are included in the supporting information (Figure S3).

### Statistical analysis

Significant differences between 10 young and 10 old animals were determined by one-tailed and unpaired t-Test using n = 10 and alpha of 0.05. One-tailed test was used, because it is known from literature, that several non-enzymatic PTMs increase in aging. The unpaired t-Test was used, because of unequal variances of young and old subgroups as indicated by F-Test. The normal distribution was checked by Shapiro-Wilk Normality Test. A single analytical replicate from every biological replicate was used, because biological variability was much higher than the analytical error.

## Supplementary information

Supplementary Information.

## Data Availability

We declare that all the data supporting the findings of this study are available within the paper and the supplementary information files.
