# Supplementary material for: Comprehensive analysis of posttranslational protein modifications in aging of subcellular compartments
Source: Sci Rep. 2020 May 5;10:7596. doi: 10.1038/s41598-020-64265-0 (PMC7200742; doi:10.1038/s41598-020-64265-0)
Supplement: Supplementary file 1 — Supplementary Information. [file 41598_2020_64265_MOESM1_ESM.pdf]

Supporting information for

Comprehensive analysis of posttranslational protein modifications in aging of subcellular compartments

Tim Baldensperger,[1] Michael Eggen,[1] Jonas Kappen,[1] Patrick R. Winterhalter,[2] Thorsten Pfirrmann[3] and Marcus A. Glomb[1, *]

[1] Institute of Chemistry, Food Chemistry, Martin-Luther-University Halle-Wittenberg, Kurt-Mothes-Str. 2, 06120 Halle/Saale, Germany

[2] Clinic for Heart Surgery, Martin-Luther-University Halle-Wittenberg, Ernst-Grube Str. 40, 06120 Halle/Saale, Germany

[3] Institute of Physiological Chemistry, Martin-Luther-University Halle-Wittenberg, Hollystr. 1, 06114 Halle/Saale, Germany

* To whom correspondence should be addressed (e-mail marcus.glomb@chemie.uni-halle.de, Tel. +49 345 552-5784, Fax. +49 345 552-7341)

Figure S1. Full-length blotting images of fractionation control. S-2

Figure S2. Structural formulas of analytes. S-3

Figure S3. Chromatographic separation of analytes by HPLC-MS/MS. S-4

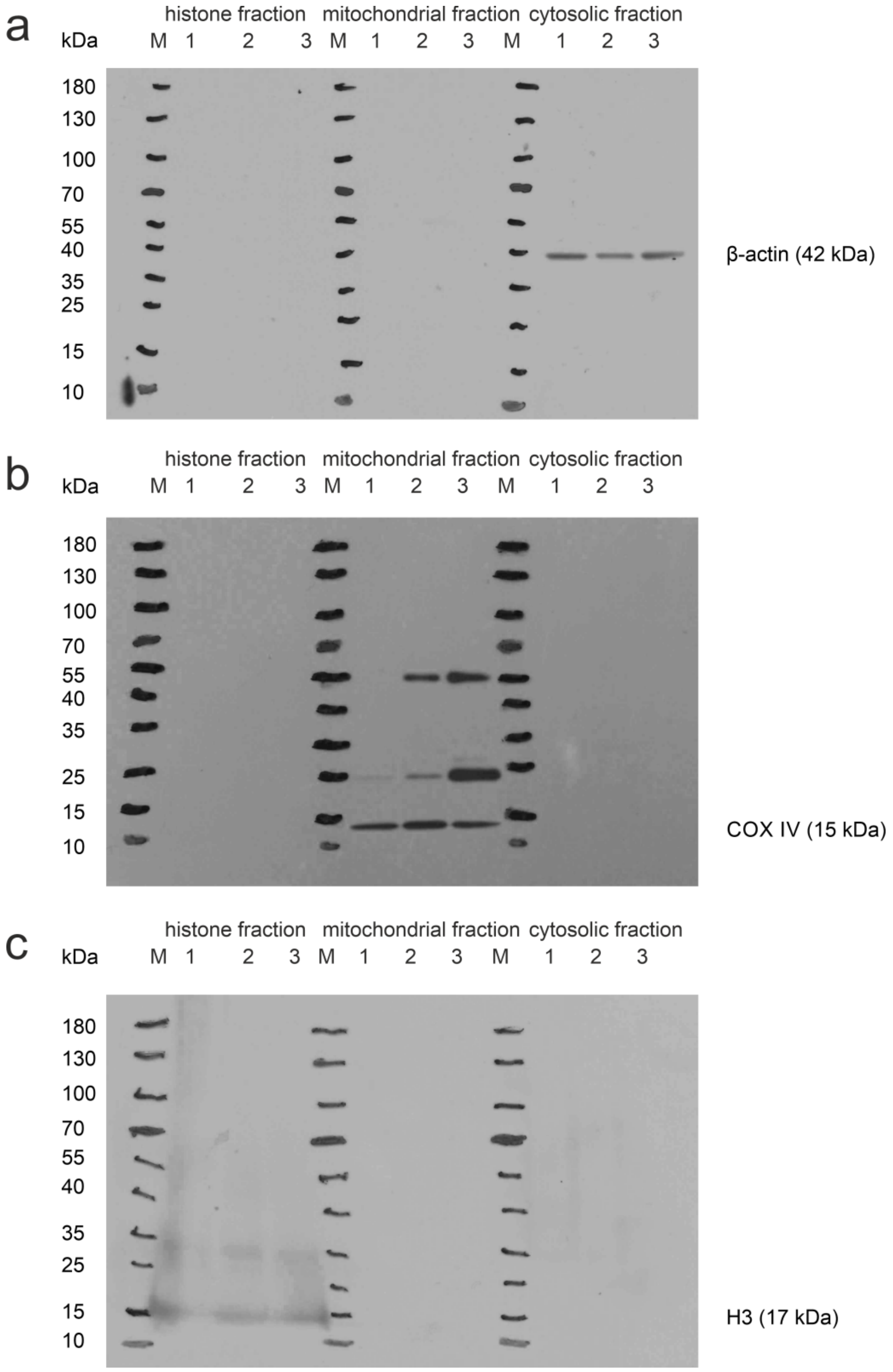

Figure S1. Full-length blotting images of fractionation control. Antibodies against cytosolic β-actin (**a**), mitochondrial COX IV (**b**), and histone H3 (**c**) were used.

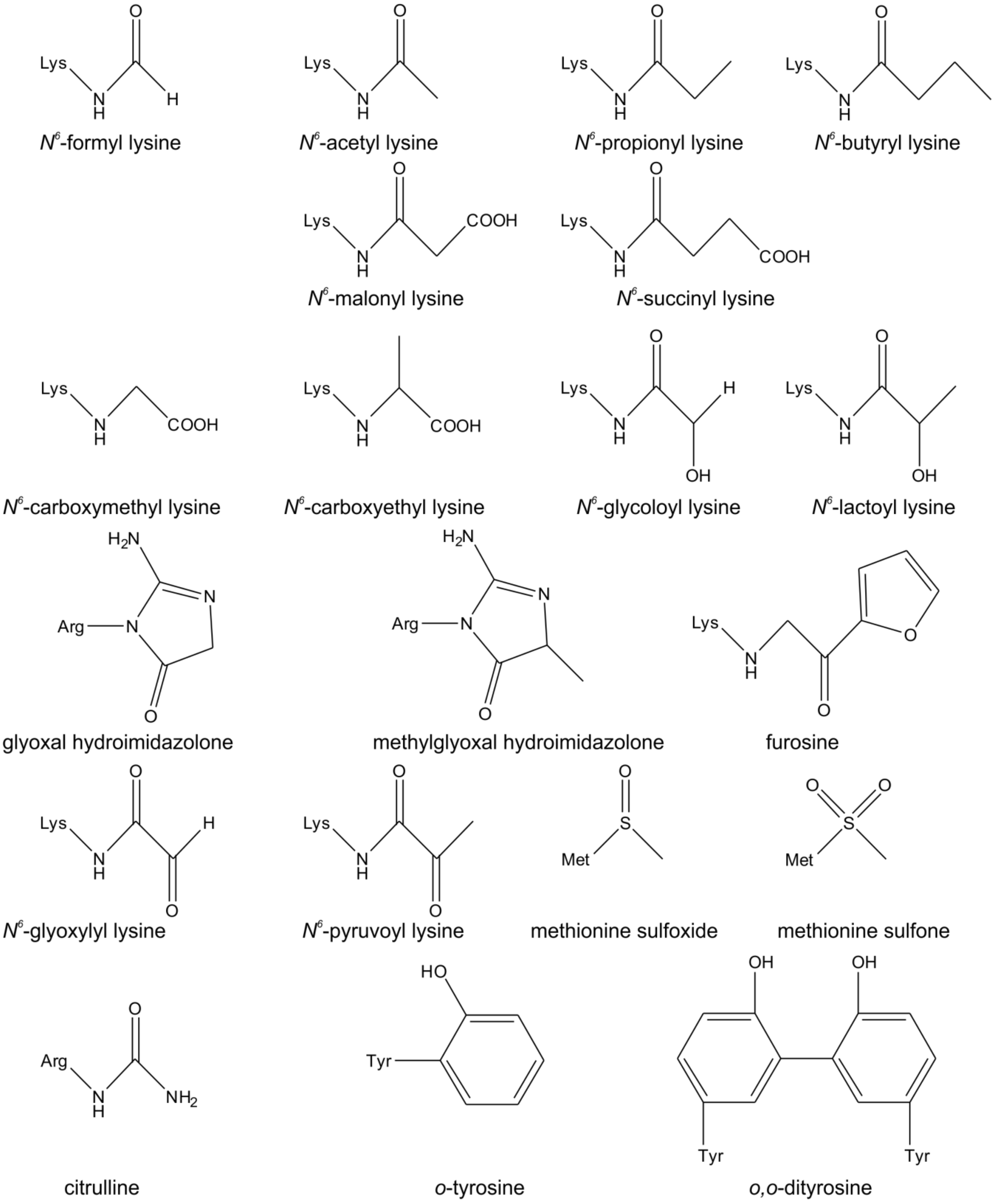

Figure S2. Structural formulas of analytes.

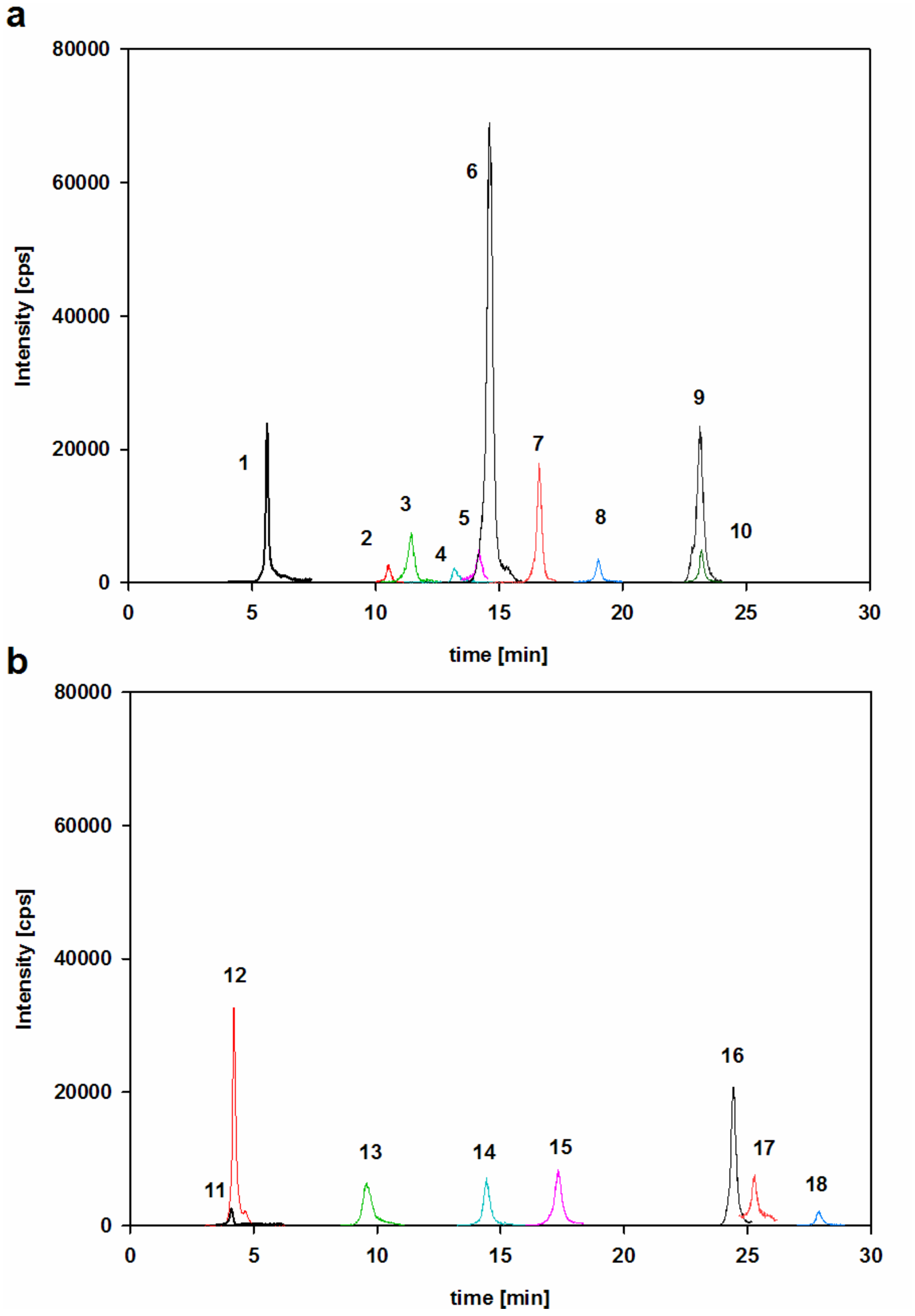

Figure S3. Chromatographic separation of analytes by HPLC-MS/MS in enzymatic (**a**) and acid (**b**) hydrolysates (**1** citrulline; **2** N^6-glycoloyl/glyoxylyl lysine; **3** N^6-formyl lysine; **4** N^6-malonyl lysine; **5** N^6-lactoyl/pyruvoyl lysine; **6** N^6-acetyl lysine; **7** N^6-succinyl lysine; **8** N^6-propionyl lysine; **9** methylglyoxal hydroimidazolone; **10** N^6-butyryl lysine; **11** methionine sulfone; **12** methionine sulfoxide; **13** N^6-carboxymethyl lysine, **14** N^6-carboxyethyl lysine, **15** glyoxal hydroimidazolone; **16** furosine; **17** *o*-tyrosine; **18** *o*,*o*-dityrosine).
